# Clinical and Immunological Outcomes in High-Risk Resected Melanoma Patients Receiving Peptide-Based Vaccination and Interferon Alpha, With or Without Dacarbazine Preconditioning: A Phase II Study

**DOI:** 10.3389/fonc.2020.00202

**Published:** 2020-03-06

**Authors:** Francesca Urbani, Virginia Ferraresi, Imerio Capone, Iole Macchia, Belinda Palermo, Carmen Nuzzo, Angela Torsello, Patrizio Pezzotti, Diana Giannarelli, Anna Fausta Pozzi, Mariano Santaquilani, Paolo Roazzi, Silvia Bastucci, Caterina Catricalà, Antonia La Malfa, Giuseppe Vercillo, Novella Gualtieri, Carla Buccione, Luciano Castiello, Francesco Cognetti, Paola Nisticò, Filippo Belardelli, Federica Moschella, Enrico Proietti

**Affiliations:** ^1^Department of Oncology and Molecular Medicine, Istituto Superiore di Sanità, Rome, Italy; ^2^Medical Biotechnology and Translational Medicine, Tor Vergata University, Rome, Italy; ^3^Department of Medical Oncology 1, IRCCS Regina Elena National Cancer Institute, Rome, Italy; ^4^Unit of Tumor Immunology and Immunotherapy, Department of Research, Advanced Diagnostics and Technological Innovation, IRCCS Regina Elena National Cancer Institute, Rome, Italy; ^5^Department of Infectious Disease, Istituto Superiore di Sanità, Rome, Italy; ^6^Biostatistical Unit, IRCCS Regina Elena National Cancer Institute, Rome, Italy; ^7^Hospital Pharmacia, IRCCS Regina Elena National Cancer Institute, Rome, Italy; ^8^Computer Control and Management, Istituto Superiore di Sanità, Rome, Italy; ^9^Health Technology Assessement, Istituto Superiore di Sanità, Rome, Italy; ^10^Clinical Trial Center, IRCCS Regina Elena National Cancer Institute, Rome, Italy; ^11^Oncological Dermatology, San Gallicano Hospital, Rome, Italy; ^12^Clinical Pathology, IRCCS Regina Elena National Cancer Institute, Rome, Italy; ^13^FAST—Istituto Superiore di Sanità, Rome, Italy; ^14^Institute of Translational Pharmacology, CNR, Rome, Italy

**Keywords:** immunotherapy, melanoma, combination therapy, chemotherapy, drug repurposing, interferon-α

## Abstract

Clinical studies based on novel rationales and mechanisms of action of chemotherapy agents and cytokines can contribute to the development of new concepts and strategies of antitumor combination therapies. In previous studies, we investigated the paradoxical immunostimulating effects of some chemotherapeutics and the immunoadjuvant activity of interferon alpha (IFN-α) in preclinical and clinical models, thus unraveling novel rationales and mechanisms of action of chemotherapy agents and cytokines for cancer immunotherapy. Here, we carried out a randomized, phase II clinical trial, in which we analyzed the relapse-free (RFS) and overall survival (OS) of 34 completely resected stage III–IV melanoma patients, treated with peptide-based vaccination (Melan-A/MART-1 and NY-ESO-1) in combination with IFN-α2b, with (arm 2) or without (arm 1) dacarbazine preconditioning. All patients were included in the intention-to-treat analysis. At a median follow-up of 4.5 years (interquartile range, 15.4–81.0 months), the rates of RFS were 52.9 and 35.3% in arms 1 and 2, respectively. The 4.5-year OS rates were 68.8% in arm 1 and 62.7% in arm 2. No significant differences were observed between the two arms for both RFS and OS. Interestingly, the RFS and OS curves remained stable starting from 18 and 42 months, respectively. Grade 3 adverse events occurred in 5.9% of patients, whereas grade 4 events were not observed. Both treatments induced a significant expansion of vaccine-specific CD8^+^ T cells, with no correlation with the clinical outcome. However, treatment-induced increase of polyfunctionality and of interleukin 2 production by Melan-A–specific CD8^+^ T cells and expansion/activation of natural killer cells correlated with RFS, being observed only in nonrelapsing patients. Despite the recent availability of different therapeutic options, low-cost, low-toxic therapies with long-lasting clinical effects are still needed in patients with high-risk resected stage III/IV melanoma. The combination of peptide vaccination with IFN-α2b showed a minimal toxicity profile and resulted in encouraging RFS and OS rates, justifying further evaluation in clinical trials, which may include the use of checkpoint inhibitors to further expand the antitumor immune response and the clinical outcome.

**Clinical Trial Registration:** https://www.clinicaltrialsregister.eu/ctr-search/search, identifier: 2008-008211-26

## Introduction

In the last decade, cancer immunotherapy has registered an impressive progress, mostly due to the clinical use of checkpoint inhibitors (CPIs), which showed long-term responses in a large variety of tumors. Because of its high immunogenicity, melanoma was the first cancer type in which CPIs were approved in metastatic (1) as well as in high-risk resected patients (2). Nevertheless, a subset of patients remains unresponsive to this therapy because of primary or secondary resistance (3). Further advances in cancer immunotherapy can only stem from a better understanding on how CPIs can be combined with additional treatments, including cancer vaccines (4).

In the history of cancer immunotherapy, many research efforts have been devoted to the development of active immunization strategies against tumor-associated antigens (TAAs), taking advantage of shared as well as neoantigens (5), with alternate cycles of optimism and discouragement. One main research challenge is how to increase the antitumor immune response to TAAs by using selected cytokines and/or drugs acting as effective immune adjuvants.

A long-standing preclinical work from our institution and other research groups had deepened our understanding for the basic mechanisms of the combined treatment of immunotherapy with chemotherapy and/or type I interferons (IFN-I) (6–8). Of note, certain chemotherapeutics (such as alkylating agents), given at defined dose and timing, may augment lymphocyte proliferation (9), reduce the number of regulatory T cells (10–12) and the expression of PD-1 on CD8^+^ T cells, favor T helper 1 (T_H_1) and T_H_17 responses (11, 12), activate polyfunctional T helper cells (13), promote tumor infiltration by T cells (14), and reset dendritic cell (DC) homeostasis (15). Remarkably, type I IFN gene signature has been demonstrated in animal models, as well as in cancer patients following administration of alkylating agent (16–18).

Interferon (IFN)-α is a cytokine belonging to the IFN-I family and endowed with pleiotropic effects, including DC development/activation (19, 20), T_H_1 cell differentiation, T cell memory turnover, and natural killer (NK) cell activation (21, 22). INF-α is the cytokine with the longest record of clinical use. For many years, the antitumor effects observed in patients with certain hematological malignancies (hairy cell leukemia and chronic myeloid leukemia) and solid tumors (including melanoma and renal cancer) contributed in maintaining a great interest of the scientific community, patients, and media on IFN-α. Today, the use of IFN-α has been largely replaced by new drugs (including targeted therapies), thought to be less toxic and more selective for cancer cells. Of note, IFN-I were used in cancer patients when their mechanisms of action were still largely unknown, as either conventional cytostatic drugs or nonspecific biological response modifiers. They were generally utilized at high dosages and administered continuously, assuming that such treatment regimens could result in more potent antitumor effects. Specific biological activities subsequently ascribed to IFN-I have poorly been considered for clinical use. As an example, an ensemble of data demonstrated that the interaction of IFN-α with specific types of immune cells, such as DC, is strictly instrumental for the induction of antitumor effects (21, 23, 24). Based on these premises, IFN-α has been used in a few clinical studies as a vaccine adjuvant in infective (25) as well as neoplastic diseases [reviewed in Rizza et al. (21)]. A pilot study showed that in stage IV advanced melanoma patients the vaccination with Melan-A/MART-1 (Melan-A) and gp100 peptides combined with low-dose IFN-α resulted in enhanced specific CD8^+^ T cells and monocyte/DC precursor activation (26). A subsequent phase I/II clinical study was conducted by our group in stage III/IV melanoma patients following surgery to evaluate the safety and immunogenicity of peptide-based vaccination with Melan-A and gp-100 in combination with low-dose IFN-α, preceded or not by a single administration of dacarbazine (DTIC) (16). Remarkably, three of five high-risk patients treated with DTIC plus IFN-α plus vaccination are up to now disease-free after more than 10 years (16). The triple combination proved safe and well tolerated and capable of inducing higher specific CD8^+^ T cell responses than vaccination plus IFN-α alone. In responder patients, we found a progressive enhancement of the T cell receptor (TCR) repertoire diversity in highly avid Melan-A–specific CD8^+^ T cells (27), accompanied by serine/threonine kinase (AKT)-activation (28).

In light of our results, we aimed to carry out an open-label, randomized, phase II trial on resected stage III, IVM1a, and IVM1b melanoma patients. Since in our previous phase I trial the immune responses to gp100 were much weaker than those to Melan-A (16), we replaced gp100 with the cancer-testis antigen NY-ESO-1, which represents a promising candidate for vaccine-based therapy given its ability to induce both cellular and humoral immune responses (29). The trial was designed to evaluate (a) whether peptide-based vaccination combined with IFN-α could improve relapse-free survival (RFS) and overall survival (OS) with respect to literature estimates available at the time of the study design; (b) whether preconditioning with DTIC could further increase the clinical outcome; (c) whether the immune response could predict the time to relapse; and (d) safety and tolerability of the investigated treatment approach.

## Materials and Methods

### Patients and Enrollment

This study (EudraCT no. 2008-008211-26) was sponsored and coordinated by Istituto Superiore di Sanità (ISS, Rome, Italy) and was conducted in accordance with the International Conference on Harmonization E6 Guidelines for Good Clinical Practice and the Declaration of Helsinki. Patients were enrolled at Regina Elena National Cancer Institute (IRE) (Rome, Italy), after having signed an informed consent form approved by the IRE Ethical Committee. Patients with histologically confirmed stage III or IV (M1a or M1b) melanoma, according to the 2002 modified American Joint Commission on Cancer (AJCC) staging system, underwent surgical resection of nodal or metastatic disease. Inclusion criteria included histologically confirmed stage III or IV (M1a or M1b) melanoma; surgical resection of nodal or metastatic disease; no evidence of disease (NED), as assessed by computed tomography (CT) scan performed within 30 days before therapy; HLA-A*0201 positivity; age 18 years or older; adequate renal, hepatic, and hematologic functions; Eastern Cooperative Oncology Group (ECOG) score 0–1; and life expectancy of at least 6 months. Exclusion criteria included current or a previous diagnosis of carcinoma within 5 years; concomitant or prior chemotherapy, immunotherapy, or radiotherapy (within 4 years); severe cardiovascular disease; concomitant immunosuppressant therapy; active autoimmune disease; active or chronic infection (including human immunodeficiency virus, hepatitis C and B viruses); pregnancy; and breastfeeding. Patients were required to have a CT scan performed within 30 days before initiation of therapy, showing NED. Patients' characteristics are detailed in Supplementary Table S1.

### Vaccine

Melan-A_26−35_ (A27L) (ELAGIGILTV) and NY-ESO-1_157−165_ (SLLMWITQC) GMP-grade peptides were produced by Polypeptide Laboratories (Strasbourg, France) and emulsified with Montanide ISA-51 (Seppic, Milan, Italy) using a two-syringe method. The emulsion was obtained by using rubber/silicone-free syringes (B. Braun, Melsungen, Germany) and flexible connector devices specifically designed by Know Medical (Viadana, Italy).

### Treatment

**Figure 1:**
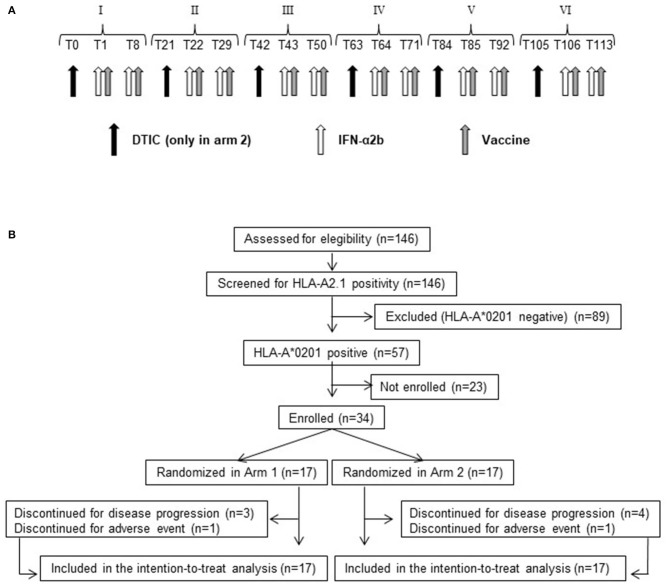
Treatment schedule and CONSORT flow diagram. **(A)** Roman numbers indicate the cycle of treatment. Tn indicates the day after beginning of treatment. Black arrow, dacarbazine (DTIC) intravenous infusion. White arrow, IFN-α2b subcutaneous injection. Gray arrow, vaccine (Melan-A/MART-1 and NY-ESO-1 peptides emulsified with Montanide ISA-51) intradermal injection. **(B)** Flow diagram showing the progress of patients throughout the trial.

In arm 1, patients received the vaccine intradermally in combination with 6 MU IFN-α2b subcutaneously (IntronA® Schering-Plough Corporation, Kenilworth, NJ, USA). Each peptide and 3 MU IFN-α2b were injected in close but separate sites near local lymph nodes in right or left alternating arms or legs. The immunization regimen consisted of six cycles (every 21 days) of two vaccine doses (7 days apart) (Figure 1A). Arm 2 patients received the same treatment of arm 1 patients, preceded (1 day before each vaccination cycle) by an intravenous infusion of 800 mg/m^2^ DTIC (Deticene; Sanofi–Aventis Groupe, Paris, France) (Figure 1A).

### Study Design

This was a single-center, open-label, randomized phase II study with the objective of gaining preliminary information regarding RFS and OS. The primary endpoint of the study was to assess whether the combination of vaccination and IFN-α2b, with or without DTIC, could increase the RFS of resected stage III/IV melanoma patients, with respect to literature estimates at the time of trial design (30). A single-stage design, as described by A'Hern (31), was used to calculate the sample size. For each arm, a sample size of 24 patients was considered sufficient to give an 80% probability of rejecting a 1-year distant metastases-free (or death-free) rate of 60% with an exact 10% one-sided significance test when the true response rate is 80%. Secondary endpoints were OS, safety and tolerability of the treatment, and immune responses analysis.

### Randomization

Randomization was performed by a computer-generated random list, with block restriction of four. The list was hidden to the clinical center.

### Patients Follow-Up

Patients' clinical status was monitored before, during, and after treatment. Complete blood count and full chemistry panel were done pretreatment and before each vaccination cycle. Total body CT scan was performed every 4 or 6 months according to the stage of disease. Locoregional lymph node ultrasound was carried out every 4–6 months. All data were recorded in electronic case report forms (eCRF), designed by the information technology service at ISS.

### Safety

Safety was evaluated by assessing incidence, severity, and nature of adverse events and graded according to NCI Common Terminology Criteria for Adverse Events (CTCAE) v4.0. The association of adverse events with treatment was determined by physicians. Adverse events were recorded in eCRF.

### Immune Response Monitoring

Blood was collected at different time points before, during (21, 42, 63, 84, 92, and 105 days), and after (4 and 6 months) treatment. Peripheral blood mononuclear cells (PBMCs) were isolated and frozen as described in (32). MIATA and MIANKA guidelines (http://miataproject.org/miata-guidelines/final-guidelines-2/) were followed to implement the data quality level of flow cytometry assays. Live and dead cells were discriminated by trypan blue exclusion method, and samples showing viability less than 70% were not further processed. Current immunological monitoring uses advanced technologies that allow the evaluation of many parameters on a small number of cells (33). The flow cytometer available when the study was performed (FACSCanto) allowed the analyses of a limited number of parameters. Considering also the low number of cells obtained by patients, we focused our analyses on specific T cell and NK cell responses as detailed below. The FACS analysis was performed on total PBMCs (1 × 10^6^ cells) or magnetically sorted CD8^+^ T cells (Miltenyi Biotech, Bergisch Gladbach, Germany), both *ex vivo* and after a short-term *in vitro* sensitization with Melan-A and NY-ESO peptides, by staining with phycoerythrin (PE)-labeled HLA-A*0201/peptide (Melan-A and NY-ESO-1) tetramers (Beckman Coulter, San Diego, CA, USA) (1 μg/10^6^ cells, 30 min, room temperature) and fluorescein isothiocyanate (FITC)–conjugated anti-CD8 mAb (Miltenyi Biotech) (15 min, 4°C). Background fluorescence (0.01%) was assessed by means of iTAgTM HLA class I human negative tetramers SA-PE (Beckman Coulter).

A multicolor flow cytometry–based approach was used to monitor variations in the percentages of the major lymphocyte and NK subsets before, during, and after treatment by using different antibody panels (anti-CD3, anti–IFN-γ, anti-CD107, anti-CD56, anti-CD16) and a dead/live staining kit, as detailed in Supplementary Table S2. All samples showed a viability greater than 88%, and for this reason, no sample was excluded from the analysis.

Functional analysis of vaccine-specific T cell responses was performed on cryopreserved PBMCs isolated at baseline and at different time points before, during (92 days), and after (4 months) treatment, by a previously described functional multiparameter test (34), combining surface staining for CD8 and HLA-A*0201/Melan-A tetramer with staining for the cytotoxicity surrogate marker CD107a and intracellular cytokine staining for IFN-γ, interleukin 2 (IL-2), and tumor necrosis factor α (TNF-α). Briefly, after thawing in the presence of DNase, 2 × 10^6^ PBMCs/well were stained with PE-labeled HLA-A*0201/Melan-A tetramer (0.5 μg/10^6^ cells), washed, and cultured in 96-well round-bottom plates in the presence of the costimulatory antibodies anti-CD49d and anti-CD28 (Becton Dickinson, San Jose, CA, USA), for 6 h at 37°C in a 5% CO_2_ incubator, in RPMI medium (Life Technologies, Gibco BRL, Grand Island, NY, US) added with 2% human serum (Euroclone, Pero, Italy), HEPES, penicillin, streptomycin, nonessential amino acids, l-glutamine, and DNase I (10 U/mL). Staphylococcal enterotoxin B (SEB; Sigma-Aldrich, Munich, Germany) (2 μg/mL) was used as positive control. During the incubation, PBMCs were stained with FITC-labeled anti-CD107a. To inhibit cytokine secretion and lysosome acidification, brefeldin A (Golgi Plug) and monensin (Golgi stop) (Becton Dickinson) were added after the first hour of incubation. After 6 h, 2 mM EDTA was added to each well and cells were incubated for 15 min. Cells were surface stained with PE/Cy7-conjugated anti-CD8 mAb (30 min at 4°C) and then washed, fixed, and permeabilized with BD intrasure kit (BD Biosciences, San Jose, CA, USA) and stained intracellularly with an antibody cocktail containing fluorescently labeled mAbs directed against IFN-γ, IL-2, and TNF-α. Fluorochromes, mAb clones, and manufacturers are detailed in Supplementary Table S2. The gating strategy is described in Supplementary Figure S1.

Natural killer cell effector functions were determined in a single-cell assay using CD107a mobilization assay and IFN-γ production. Cells were stimulated with K562 cells at 25:1 effector/target ratio or PMA (1.25 ng/mL) and ionomycin (1μg/mL) (Sigma-Aldrich, St. Louis, MO, USA) as positive control. In brief, 1 × 10^6^ thawed PBMCs were cultured in U-bottom plates for 4 h at 37°C cells in the presence of monensin (Golgi Stop; BD Biosciences) and brefeldin A (Golgi Plug; BD Biosciences). Spontaneous degranulation (CD107a^+^ percentage) and IFN-γ secretion were determined in the absence of targets and stimuli. Fluorescein isothiocyanate–labeled anti-CD107a was added at the beginning of incubation. After culturing, cells were labeled for 20 min at 4°C with anti-CD16, anti-CD56, and anti-CD3. Cells were then washed, lysed, and permeabilized with BD intrasure kit (BD Biosciences) and stained with anti–IFN-γ. A LIVE/DEAD Fixable Near-IR Dead Cell Stain Kit (Molecular Probes, Eugene, OR, USA) was used to determine the viability of cells prior to surface and intracellular staining. FcR blocking (BD Biosciences) was also included in order to avoid nonspecific staining of monoclonal antibodies to FcγRIII. The gating strategy for NK and NKT cells is described in Supplementary Figure S2.

Data acquisition was performed using a FACSCanto instrument (BD Biosciences) and analyzed by FACS DIVA or FlowJo version 10 (Tree Star, Ashland, OR, USA) or Kaluza (Beckman Coulter, Brea, CA, USA) software. For each sample, all labeled cells (up to 1,300,000 events) were acquired in order to give statistical significance to very low expressed or even rare populations. Abnormal or manifestly artifactual acquired samples were not further analyzed (e.g., light scatter or any fluorescence abnormal profile).

### Statistical Analysis

The sample size was calculated for each treatment arm using a single-stage design as described by A'Hern (31). Based on the EORTC 18991, the expected 1-year RFS rate of untreated stage III resected melanoma patients was 60% (30). For each arm, a sample size of 24 patients was considered sufficient to give an 80% probability of rejecting the null hypothesis, with an exact 10% one-sided significance test when the true response rate is 80%.

Relapse-free survival and OS were evaluated by intention-to-treat analysis including all randomized patients.

Relapse-free survival was measured from the date of randomization until the date of relapse or death from any cause, and OS was measured from the date of randomization until death from any cause (Supplementary Table S1). For patients who were disease-free or alive at the time of data cutoff or for patients lost to follow-up, survival was censored on the last date of follow-up. The Kaplan–Meier method was used to estimate median survival, RFS, and OS distributions and the 95% confidence interval (CI) of these estimates [1.96 times the standard error (SE) in each direction], where the SE was computed with the Greenwood formula. The Brookmeyer and Crowley method was used to calculate the 95% CI of median RFS and OS. Stratified log-rank test, at a two-sided α level of 0.05, was used to compare distributions of OS and RFS between treatment arms.

A χ^2^ test or Fisher exact test was used to compare different groups of patients for the analysis of toxicity.

Regarding immunological markers, comparisons between arm 1 vs. arm 2 and relapsing vs. disease-free patients were performed by independent nonparametric Mann-Whitney *U*-test.

Wilcoxon nonparametric test for paired sample was used to analyze differences between time points.

Statistical analysis was performed using IBM-SPSS processor v25 (IBM Corporate New York, NY, USA) and STATA (StataCorp LLC 4905 Lakeway Drive, Texas, USA).

## Results

### Patients' Characteristics and Treatment

From February 23, 2010, to August 10, 2012, 146 stage III, IVM1a, IVM1b melanoma patients, undergoing surgical resection of metastatic or nodal lesions and complying with all other eligibility criteria, were screened for HLA-A*0201 expression. A total of 57 patients were found HLA-A2 positive, and 34 were enrolled and randomly allocated to either arm 1 (17 patients) or arm 2 (17 patients) (Figure 1B and Supplementary Table S1).

Patients allocated in arm 1 were treated with peptide-based vaccination [Melan-A:26-35(27L) and NY-ESO-1:157-165, emulsified with Montanide ISA-51]. The immunization regimen consisted of six cycles (every 21 days) of two vaccine doses (7 days apart), administered in combination with 6 MU IFN-α2b (Figure 1A). Arm 2 patients received the same treatment of arm 1, preceded (1 day before each vaccination cycle) by DTIC (800 mg/m^2^) (Figure 1A).

Table 1 and Supplementary Table S1 show demographic and clinical characteristics of patients. Overall, 1 patient (3%) had stage IIIA, 4 (12%) had stage IIIB, 17 (50%) had stage IIIC, 9 (27%) had stage IVM1a, and 2 (8%) had stage IVM1b disease. Stage, age, gender, ethnicity, ECOG status, and LDH values were not significantly different between the two arms (Table 1). All patients showed an ECOG performance status of 0. Median LDH level before vaccination was 305 U/L (range, 197–433 U/L), falling within the reference range values.

**Table 1:** Patient demographic and baseline characteristics.

**Characteristics**	***n***^**a**^ **(%) or Median (range)**		
	**All**	**Arm 1**	**Arm 2**
Median age (range), years	52 (23–80)	55 (40–80)	46 (23–73)
**Sex**, ***n*** **(%)**			
Male	21 (62)	11 (65)	10 (59)
Female	13 (38)	6 (35)	7 (41)
**Ethnicity**, ***n*** **(%)**			
White	34 (100)	17 (100)	17 (100)
**ECOG performance status**, ***n*** **(%)**			
0	34 (100)	17 (100)	17 (100)
**AJCC stage**, ***n*** **(%)**			
IIIa	1 (3)	1 (6)	0 (0)
IIIb	4 (12)	2 (12)	2 (12)
IIIc	17 (50)	8 (47)	9 (53)
IV M1a	9 (27)	5 (29)	4 (24)
IV M1b	3 (8)	1 (6)	2 (12)
Median LDH,^b^ range (U/L)	305 (197–433)	309 (197–433)	300 (260–432)

a *Data are n or median, as indicated. Percentage (%) or range in brackets*.

b *LDH normal range, 220–480 U/L*.

Seven patients (20.6%) (three in arm 1 and four in arm 2) discontinued treatment because of disease progression. Two patients (5.9%) (one in arm 1 and one in arm 2) discontinued treatment because of adverse events (Figure 1B).

### Clinical Results

On November 2018, clinical data cutoff date, the median follow-up duration was 55.1 months (4.5 years) (interquartile range, 15.4–81.0 months).

**Figure 2:**
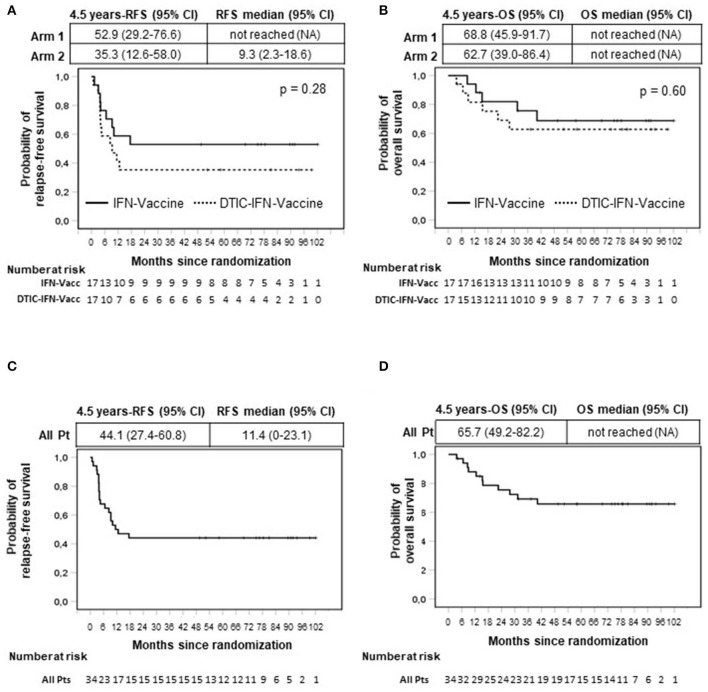
Intention-to-treat analysis of relapse-free (RFS) **(A,C)** and overall survival (OS) **(B,D)** by Kaplan–Meier method. All enrolled patients were included in the analysis (*n* = 34). Months are calculated since time of randomization. Arm 1 patients (Pt) (*n* = 17) were treated with vaccination with Melan-A and NY-ESO-1 peptides (Vaccine) and interferon-α2b (IFN). Arm 2 patients (*n* = 17) received the same treatment of arm 1 patients with the addition of dacarbazine (DTIC) pretreatment. **(A,B)** Comparison between arms. **(C,D)** All patients (*n* = 34). *p* value by log-rank test.

The intention-to-treat analysis is shown in Figure 2 for both treatment arms (34 patients). In both treatment arms, all recurrences were observed within 18 months following randomization.

Of note, the probability of relapse was mostly concentrated in the first year (50%; 95% CI, 34.7–67.6%), although it became very low after this period and equal to zero after 17 months (Figure 2A).

Eighteen months following randomization, 9 of 17 patients treated with vaccination and IFN-α2b (arm 1) were still relapse-free and alive, and remarkably, no further relapses were observed thereafter. Therefore, the RFS rate was 52.9% (95% CI, 29.2%−76.6%) at 1.5 years and remained the same at 4.5 years (median follow-up duration), when five or more patients were still under observation and even beyond (fewer than 5 patients under observation) (Figure 2A). The median RFS was not reached (Figure 2A).

Of the 17 patients receiving preconditioning DTIC before vaccination (arm 2), six had NED at 18 months and remained disease-free until their last follow-up. The 4.5-year RFS rate was 35.3% (95% CI, 12.6%−58.0%), and the median RFS of this patient cohort was 9.3 months (95% CI, 2.3%−18.6%) (Figure 2A).

Although the sample size was not dimensioned to compare the two treatment arms, no significant differences were observed between them (Figure 2A). On the whole, 15 of 34 patients were still relapse-free at their last follow-up, and the cumulative 4.5-year RFS rate of all treated patients was 44.1% (95% CI, 27.4%−60.8%) (Figure 2C). The median RFS of all treated patients was 11.4 months (95% CI, 0–23.1 months) (Figure 2C).

After 4.5 years, some patients were lost to follow-up (≤12 patients at risk). Interestingly, at 6 years following randomization, 11 of 11 patients under observation were disease-free. Of particular relevance, a stage IV M1a patient (patient 4, arm 1) was NED up to 8 years (Figure 2C and Supplementary Table S1). Among patients who relapsed, one patient (patient 011) who relapsed 13 months after randomization had surgical removal of relapsed tumor and remained disease-free until the last follow-up (7.6 years). Patient 024 (arm 2), who relapsed 4 months after randomization, underwent a second surgery and relapsed again 22 months later. However, this patient (patient 024) displayed a complete response after treatment with dabrafenib and trametinib, which lasted until the last follow-up (4 years) (Supplementary Table S1).

The OS (secondary endpoint of the study) is shown in Figures 2B,D. In the cohort of patients treated with vaccination and IFN-α2b, 5 of 17 patients died. As shown in Figure 2B and Supplementary Table S1, the last death was observed at 40 months. The 4.5-year OS rate was 68.8% (95% CI, 46.1%−91.5%), and the OS curve remained stable later on. The median OS has not been reached. The OS rate at 4.5 years for patients treated with the combination of DTIC plus vaccination plus IFN-α2b was 62.7% (95% CI, 39.0%−86.4%) and remained stable thereafter (six deaths were reported). The median OS was not reached (Figure 2B). No statistically significant difference was found between the two treatment arms (Figure 2B), and the cumulative 4.5-year OS rate was 65.7% (95% CI, 49.2%−82.2%) (Figure 2D).

In Supplementary Table S1, the RFS and OS are reported for each patient along with demographic and disease characteristics.

### Safety and Toxicity

Overall, the treatment was well tolerated. Adverse events for any cause are reported in Table 2. There were neither treatment-related deaths nor grade 4 adverse events during the treatment courses. Only two patients (5.9%) presented with grade 3 adverse events: severe musculoskeletal pain (arm 1) and severe asthenia (arm 2). These adverse events lead to patients' withdrawal from the trial. Other common adverse events (mostly grade 1) were fever (68%), musculoskeletal pain (27%), asthenia (24%), nausea (29%), and vomiting (18%) (Table 2). Noticeably, most of the reported adverse events were typical side effects of IFN-α treatment (i.e., fever, musculoskeletal pain, headache) and persisted for no more than 1 day. Exceptionally, nausea and vomiting were significantly related to DTIC administration (*P* = 0.004) (Table 3).

**Table 2:** Treatment-related adverse events.

**Event**	**Grade^**a**^ 1**	**Grade 2**	**Grade 3**	**Grade 4/5**
	***n*** **(%)**			
Fever	22 (65)	1 (3)	0	0
Nausea	9 (26)	1 (3)	0	0
Musculoskeletal pain	8 (24)	1 (3)	1 (3)	0
Vomiting	6 (18)	0	0	0
Asthenia	5 (15)	3 (9)	1 (3)	0
Headache	3 (9)	0	0	0
Injection site reaction/erythema	2 (6)	0	0	0
Bradycardia	1 (3)	0	0	0
Diarrhea	1 (3)	1 (3)	0	0
Nail dyschromia	1 (3)	0	0	0
Local pain	1 (3)	0	0	0
Epigastric pain	1 (3)	1 (3)	0	0
Injection site reaction/swelling	1 (3)	0	0	0
Herpes labialis	1 (3)	0	0	0
Injection site reaction/hyperemia	1 (3)	0	0	0
Hypotension	1 (3)	0	0	0
Mild visus decrease	1 (3)	0	0	0
Loss of appetite	1 (3)	0	0	0
Agitation	1 (3)	0	0	0
Vertigo	1 (3)	0	0	0
Anemia	0	1 (3)	0	0
Abdominal pain	0	1 (3)	0	0
Neutropenia	0	1 (3)	0	0
Persistent coughing	0	1 (3)	0	0

a *Grades of adverse events were defined according to the National Cancer Institute CTCAE, version 4.0*.

**Table 3:** Nausea and vomiting in the different treatment arms.

		**Arm**		**Total**
		**1**	**2**	
Nausea and/or vomiting^a^	Not present	15	6	21
	Present	2	11	13
	Total	17	17	34

a *Nausea and/or vomiting were significantly associated to the treatment (Fisher exact test p = 0.004). Arm 1 patients were treated with vaccination and IFN-α2b. Arm 2 patients were treated with vaccination, IFN-α2b, and dacarbazine*.

### Evaluation of the Vaccine-Specific Immune Response

To evaluate whether the vaccination with Melan-A and NY-ESO-1 peptides was able to induce or increase specific CD8^+^ T cell responses and in order to characterize their functionality, peripheral blood samples were taken before, during, and after treatment in 29 patients evaluable for response (excluding patients who discontinued early the trial).

**Figure 3:**
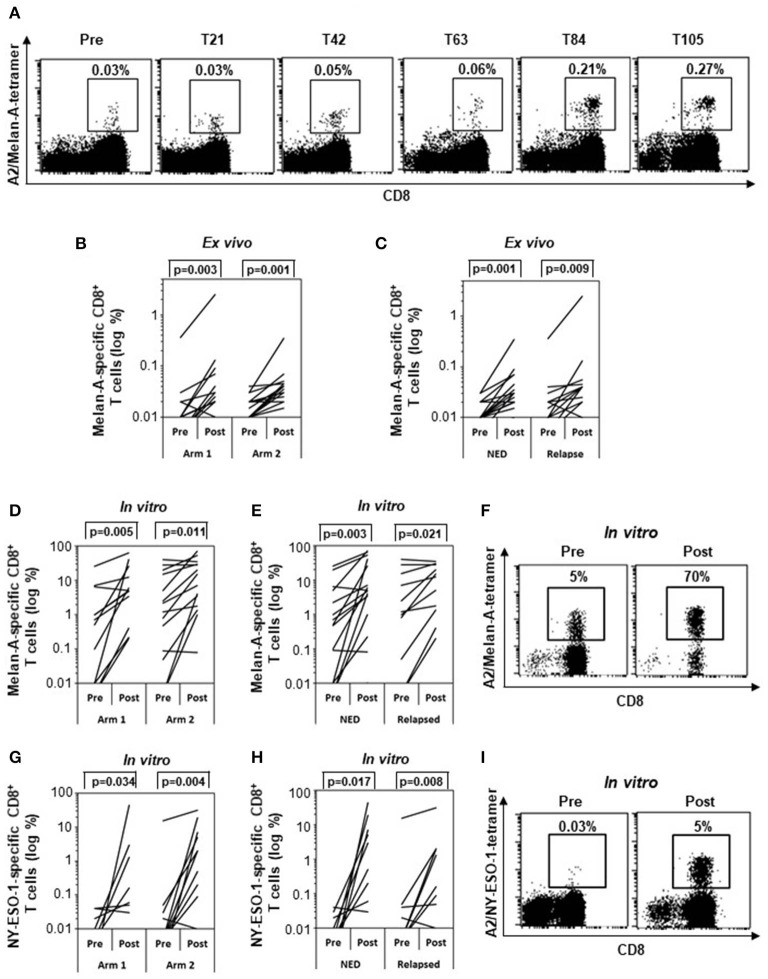
Specific immune response. Frequencies of Melan-A– **(A–F)** and NY-ESO-1–specific **(G–I)** CD8^+^ T cells analyzed by tetramer staining (*n* = 29). **(A)** Kinetic analysis of the frequency of Melan-A–specific T cells between pretreatment and posttreatment samples (T21, T42, T63, T84, T105) in one representative patient (patient 29). **(B,C)** Variation of Melan-A–specific T cell percentage between pretreatment and posttreatment *ex vivo* samples in arm 1 (*n* = 15) vs. arm 2 patients (*n* = 14) **(B)** and in patients with no evidence of disease (NED) (*n* = 16) vs. relapsed patients (*n* = 13) **(C)**. **(D,E,G,H)** Variation of Melan-A– (*n* = 28) **(D,E)** and NY-ESO-1–specific (*n* = 26) **(G,H)** T cell percentage after short term *in vitro* expansion, in arm 1 vs. arm 2 patients **(D,G)** and in patients with no evidence of disease (NED) vs. relapsed patients **(E,H)**. **(F,I)**. Representative staining (patient 09) of short term *in vitro* expansion before (pre) and after (post, T105) stimulation with Melan-A (F) and NY-ESO-1 (I) peptides. *p* values were calculated by Wilcoxon signed-rank test.

First, PBMCs were e*x vivo* analyzed by flow cytometry to assess the percentages of Melan-A/NY-ESO-1 tetramer–positive CD8^+^ T cells. NY-ESO-1–specific T cell numbers were in most cases below the level of detection (data not shown). In case of Melan-A, the kinetic of response in one representative patient (patient 29) showed that the frequency of Melan-A–specific CD8^+^ T cells doubled starting from T63 (i.e., after three treatment cycles) to reach a plateau at T84 (Figure 3A). Therefore, in all evaluable patients (*n* = 29), the T cell response was analyzed at T0 and between T84 and T105, depending on sample availability. Before treatment (pre), a low frequency (between 0.01 and 0.04%) of Melan-A–specific CD8^+^ T lymphocytes was detectable in 23 patients. One patient (patient 14) had a high spontaneous Melan-A–specific T cell response (0.36%) (Figure 3B). A significant (twofold or greater) increase of Melan-A tetramer–positive CD8^+^ T cell frequencies was observed in 20 of 29 patients analyzed (68.96%) following treatment (post) in both arms (*P* = 0.003 in arm 1 and *P* = 0.001 in arm 2) (Figure 3B). In particular, the combination of the vaccine with IFN-α2b and DTIC induced a T cell response in 12 of 15 patients (80%) (*P* = 0.001), whereas, 8 of 14 patients (57.2%) (*P* = 0.003) responded to the combination of the vaccine with IFN-α2b alone (Figure 3B). We then compared whether the response to Melan-A correlated with the patient clinical outcome. As shown in Figure 3C, a statistically significant increase of Melan-A–specific T cell frequencies was observed after treatment in both NED (81%) (*P* = 0.001) and relapsing patients (61%) (*P* = 0.009). However, the survival curves of responding and nonresponding patients were not significantly different (Supplementary Figure S3).

To determine their proliferative potential, lymphocytes were sensitized *in vitro* in the short term with Melan-A (Figures 3D–F) or NY-ESO-1 (Figures 3G–I) peptides and analyzed by tetramer staining. A significant expansion of T cells specific for both epitopes was observed in both treatment arms (Figures 3D,G) and in both NED and relapsing patients (Figures 3E,H). Staining of lymphocyte expanded by a short-term *in vitro* stimulation with Melan-A (F) and NY-ESO-1 (I) peptides is shown before (Pre) and after (Post, T105) treatment, for a representative patient (patient 09).

**Figure 4:**
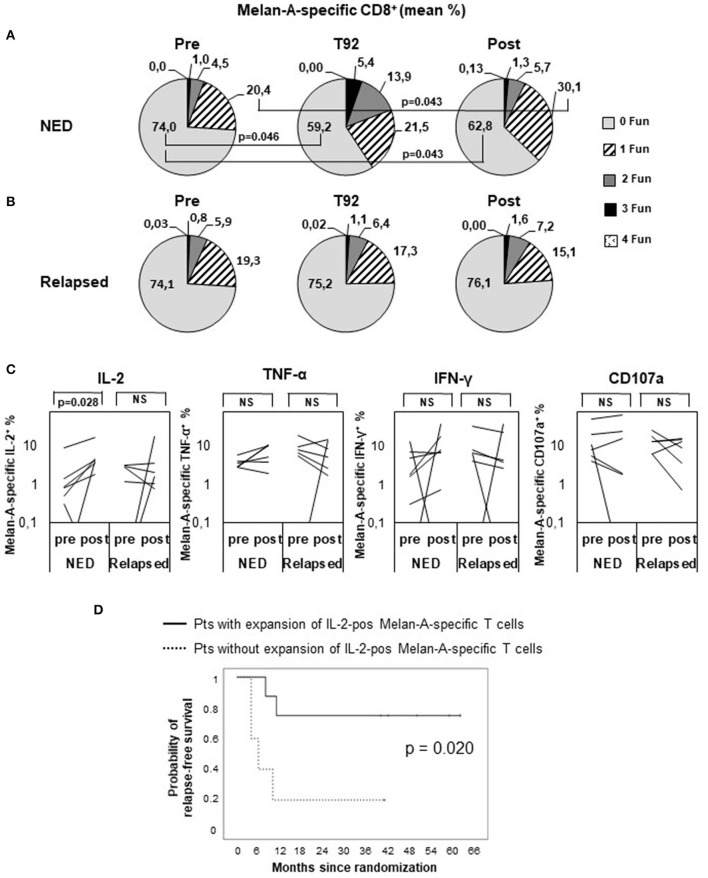
Polyfunctional analysis of Melan-A–specific CD8^+^ T cells. Peripheral blood mononuclear cells were analyzed by multiparameter flow cytometry after 5 h *in vitro* culture without peptide pulsing, followed by 1-h incubation with brefeldin A and monensin. CD8^+^ Melan-A tetramer–positive T cells were gated as shown in Supplementary Figure S1 and analyzed for their simultaneous expression of surface CD107a and intracellular IL-2, IFN-γ, and TNF-α. **(A,B)** Pie charts showing the proportion of cells expressing any combination of four (4 Fun), three (3 Fun), two (2 Fun), one (1 Fun), or zero (0 Fun) tested markers (CD107a, IL-2, IFN-γ, and TNF-α). Data are expressed as mean percentage of CD8^+^ Melan-A tetramer–positive T cells. **(A)** Patients with no evidence of disease (NED). **(B)** Patients with disease recurrence (Relapsed). *p* values by Wilcoxon signed-rank test. **(C)** Variation of Melan-A–specific T cell percentage expressing intracellular IL-2, TNF-α, IFN-γ, or surface CD107a (logarithmic scale) between pretreatment and posttreatment (4 months) in patients with no evidence of disease (NED) (*n* = 7) vs. relapsed patients (*n* = 6). *p* values by Wilcoxon signed-rank test. **(D)** Kaplan–Meier plot comparing the relapse-free survival of patients (Pts) characterized, or not, by a twofold expansion of IL-2–positive Melan-A–specific T cells in post vs. pre samples. *p* value by log-rank test.

To assess whether disease-free and relapsing patients developed a T cell response with different functionalities, we analyzed the ability of Melan-A–specific cells to simultaneously produce CD107a, TNF-α, IL-2, and IFN-γ in selected patients (16 patients with available frozen samples and showing at least 0.01% of CD8^+^ cells in both pre and post samples) (Figure 4). The gating strategy is shown in Supplementary Figure S1. Before treatment (pre), most of the Melan-A–specific cells in both NED (Figure 4A) and relapsing patients (Figure 4B) did not express any tested function (74%), ~20% of cells expressed one function, ~5% were double positive, ~1% expressed three functions, and almost none expressed four functions. Remarkably, 92 days (i.e., after nine vaccinations) and 4 months following treatment onset, only in patients who remained disease-free the Melan-A–specific cell functionality increased. In fact, the percentage of Melan-A–specific cells expressing none of the tested functions significantly diminished from the pretreatment level of 74 to 59.2% at T92 (*P* = 0.046) and 62.8% posttreatment (*P* = 0.043). The percentage of single positive cells increased from 20.4 to 30.1% at 4 months (*P* = 0.043). For double- and triple-positive cells, a trend of increase from 4.5 to 13.9% and from 1 to 5.4%, respectively, was observed at T92 (Figure 4A). Conversely, in relapsing patients, no changes in the percentages of zero-functional, monofunctional, and polyfunctional cells were observed (Figure 4B).

We then analyzed whether the modulation of any defined function (or combination of functions) correlated with the patient clinical outcome and found that the production of IL-2 by Melan-A–specific lymphocytes was significantly increased following treatment only in nonrelapsing patients (*P* = 0.028) (Figure 4C). Remarkably, the RFS curve of patients in which the percentage of IL-2^+^ Melan-A–specific cells increased following treatment was significantly (*P* = 0.020) different from the curve of patients with no expansion of these cells (Figure 4D). Interestingly, in relapsing patients, a trend of reduction of TNF-α, IFN-γ, and CD107a producing cell percentages was observed after treatment (Figure 4C), thus highlighting a different functionality of peptide-specific T cells in patients with different clinical response. A similar set of analyses was conducted to compare whether Melan-A–specific T cells had a different functionality in arm 1 and arm 2 patients, and no significant differences were found (data not shown).

### Evaluation of the Innate Immune Response

Natural killer cells have been shown to control tumor growth in particular when tumors downregulate MHC I. Because type I IFN can modulate innate immunity, including NK cells (6) by affecting their number and cytotoxic capacity (22), an in-depth analysis of the frequency, phenotype, and functional abilities of these cells was carried out. The gating strategy is shown in Supplementary Figure S2.

**Figure 5:**
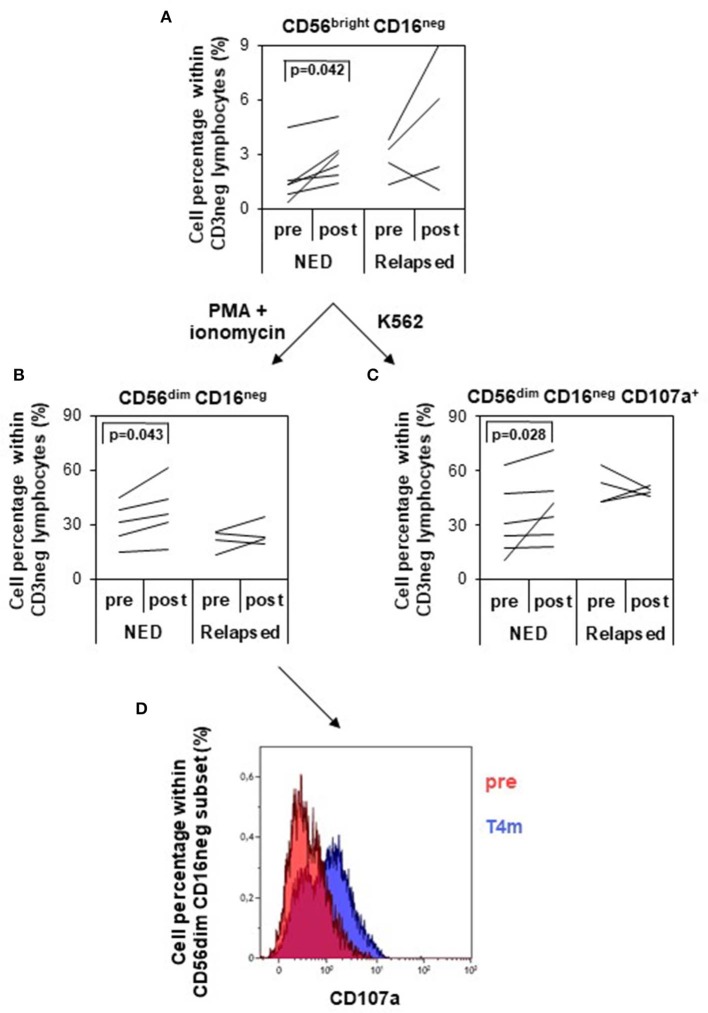
Analysis of natural killer (NK) cell subsets and of their functionality. NK cells were identified and divided into four different subsets based on the expression of CD56 and CD16 within the CD3 negative lymphocytes (gating strategy depicted in Supplementary Figure S2). **(A)** Variation of the percentage of CD56^bright^ CD16^neg^ NK cells (logarithmic scale) between pretreatment and posttreatment (4 months) in patients with no evidence of disease (NED) (*n* = 6) vs. relapsed patients (*n* = 4). **(B,C)** NK cells were *in vitro* cultured with PMA/ionomycin **(B)** or with K562 target cells **(C)**, and CD56^dim^ CD16^neg^ NK cells were analyzed for their percentage variation **(B)** and CD107a expression **(C)** pretreatment and posttreatment in NED (*n* = 6) and relapsed patients (*n* = 4). *p* values by Wilcoxon signed-rank test. **(D)** Functional analysis of CD56dim CD16neg NK cells in response to K562 target cells pretherapy and 4 months after therapy in one representative patient (patient 28). CD107a-positive cells increased after treatment.

The analysis of the frequencies of total NK (CD56^+^CD3^−^) and NK-like-T (NKT) cells (CD56^+^ CD3^+^) showed no differences between pretreatment and posttreatment time points (data not shown). However, the proportion of the different NK subsets changed following treatment. In particular, CD56^bright^CD16^neg^ NK cell subset was significantly increased after treatment in disease-free patients, whereas in relapsing patients only a trend toward increase was observed (Figure 5A). Following *in vitro* activation with PMA/ionomycin, these cells were able to differentiate toward a more mature phenotype, that is, CD56^dim^CD16^neg^ (Figure 5B) more efficiently in NED (*P* = 0.043) than in relapsing patients, and their functional ability increased following treatment only in disease-free patients. In fact, when challenged with MHC devoid target cells (K562 cells), the proportion of CD56^dim^CD16^neg^ cells expressing CD107a, as marker of degranulation, was significantly expanded (*P* = 0.028) (Figure 5C), whereas the percentage of cells expressing IFN-γ did not change (data not shown). In Figure 5D, the increase in the percentage of CD56^dim^CD16^neg^-expressing CD107a between pretreatment and 4 months after treatment is shown for one representative patient (patient 28).

## Discussion

The incidence rate of malignant melanoma is constantly increasing, reaching five cases per 100,000 persons (95% CI, 4–7) worldwide and 16 cases per 100,000 persons (95% CI, 11–20) in Western Europe in 2015 (35). Stage III–IV patients have high risk of recurrence after primary melanoma resection (36, 37). Insights into the complex relationship between the host immune response and the tumor have led to the approval of different immunotherapies to prevent recurrence in high-risk patients.

The first adjuvant treatment approved by the Food and Drug Administration (FDA) and the European Medicines Agency (EMA) for stage III patients with high risk of recurrence was high-dose IFN-α (IFN-α2b). Pegylated IFN-α2b was approved by FDA in 2011. The 4-year RFS rate in patients treated with pegylated IFN was 45.6% as compared to 38.9% in the observational group, whereas inconsistent increases in the OS were observed. The treatment was also associated to substantial toxic effects (30).

Starting in 2015, three CPIs have been approved for the adjuvant treatment of melanoma by FDA and EMA, that is, ipilimumab (38, 39), nivolumab (40), and pembrolizumab (41). Later on, the combination of the BRAF inhibitor (dabrafenib) and the MEK inhibitor (trametinib) was shown to improve survival of stage III patients with BRAF V600 mutations (42, 43) and was approved for the adjuvant treatment of melanoma. Although showing impressive antitumor effects, CPIs and kinase inhibitors are characterized, up to now, by some toxicity (especially for ipilimumab at the FDA-approved dose of 10 mg/kg) and by elevated costs, which limit their full utilization on a wide number of patients, such as resected high-risk patients. Moreover, not all patients respond to checkpoint blockade or kinase inhibitors, and not all responses are long-lasting (3). Thus, effective, low-cost, and low-toxicity therapies are still needed in melanoma, especially in the adjuvant setting; thus, it is still of great interest to investigate novel combinations of immunotherapies based on solid scientific rationales.

The present phase II study was designed to provide preliminary evidence of the efficacy of peptide-based vaccination in combination with IFN-α2b, preceded or not by DTIC, to prevent relapse of completely resected melanoma patients with high risk of recurrence (stage III/IV). When the present study began, no FDA/EMA–approved adjuvant treatment was available for stage III–IV melanoma patients. Therefore, no active control could have been used in this study.

The primary endpoint of this study was to assess whether the combination of vaccination and IFN-α2b (with or without DTIC) could increase the 1-year RFS rate from the 60% observed in the untreated control arm of a phase III study (30) to 80%. Because an interim analysis showed a 1-year RFS rate of 58.8% (95% CI, 35.5%−82.1%) in arm 1 patients and of 41.2% (95% CI, 17.9%−64.5%) in arm 2, patient enrollment was stopped at 17 patients per arm, before reaching the preplanned sample size of 24. Notably, this goal and, accordingly, the sample size were chosen based on a phase III clinical study including only stage III resected patients (30), which are characterized by a more favorable prognosis than a mixed population of stage III/IV patients. In hindsight, this goal was indeed overestimated, considering that, in a recently published phase III clinical trial, the 1-year RFS rate of resected stage III/IV patients was 60.8% in ipilimumab-treated patients and 70.5% in the nivolumab group (40).

The intention-to-treat analysis showed interesting results with regard to the clinical outcome of patients treated with melanoma peptides plus IFN-α. In fact, the 4.5-year RFS rate of patients receiving vaccination with Melan-A and NY-ESO-1 peptides in combination with IFN-α2b was 52.9%, and the 4.5-year OS rate was 68.8%. The RFS and OS curves became stable starting from 18 and 40 months, respectively, and, noticeably, remained stable up to 4.5 years (median follow-up time). After 4.5 and 6.5 years, the estimates of RFS and OS are less reliable, respectively, because fewer than five patients are at risk in one of the two arms. However, it is interesting to note that no further relapses or death were observed in the patients with longer follow-up (up to 8 years). Although comparisons between different studies should be interpreted with carefulness and despite the limited robustness of our data derived from a small phase II study, the clinical results obtained here appear to be comparable to those observed in randomized phase III studies. As a matter of fact, resected patients treated with pegylated IFN-α2b showed a 4-year RFS rate of 45.6% and an OS rate of 71% (30). In the ipilimumab trial, the 5-year RFS rate was 40.8% in the ipilimumab group vs. 30.3% in the placebo group and OS rate of 65.4% vs. 54.4 (39). In a more recent phase III trial the 3- and 4-year RFS rates were, respectively, 59 and 54% in the dabrafenib plus trametinib arm and 40 and 38% in the placebo arm, whereas the 3-year OS rate were 86% in treated patients vs. 77% in the placebo group (42, 43). Noteworthy, patients included in these three studies were all stage III, whereas those enrolled in our study were stage III (65%) and IV (35%), thus presenting a higher risk of recurrence. Promising results of recently published phase III study in stage III/IV resected patients treated with nivolumab showed improved 1-year RFS rates with respect to ipilimumab (70.5% in the nivolumab group as compared to 60.8% in the ipilimumab group) (40). Recent pembrolizumab results are also highly promising (1-year rate of RFS 75.4% vs. 61.0% in the placebo group) (41), but longer follow-up are still needed. In a small melanoma vaccine trial testing the combination of high-dose IFN-α with autologous DC, transduced with three shared/nonmutated melanoma antigens, it was observed that among the 11 stage III/IV resected patients NED at baseline, seven recurred, and four remained NED (36.3 %) up to 3 years (3.7–37.5+ months). No indications of the OS of these patients are given in the article (44).

Of note, with regard to the present study, it is worth underlining that our combination strategy was devoid of major toxic effects.

Contrary to what we expected based on results of our previous phase I clinical trial (16), pretreatment with DTIC before each vaccination cycle significantly increased neither RFS nor OS. One possible explanation for this discrepancy may rely on the different dose of IFN-α administered in the two studies, that is, 3 MU in the phase I trial (16) and 6 MU in the present phase II study. Immunodominance is a property of CD8^+^ T cell responses to viruses and vaccines, which determines the skewing of the T cell response toward a few epitopes. CD8^+^ T cells recognizing their cognate ligand were shown to inhibit the proliferation of other CD8^+^ T cells engaged with the same APC (45, 46). Based on the hypothesis that the separate administration of the two vaccine peptides could avoid their competition for MHC binding and CD8^+^ T cell cross-competition (47, 48), in the present study we injected Melan-A and NY-ESO-1 into distant sites, and because each peptide administration was associated with the nearby injection of 3 MU IFN-α2b, the total IFN-α dosage was doubled. As we previously observed that DTIC itself induces an IFN-I–related gene signature (16), possibly responsible for its immunomodulatory properties, we suppose that doubling the dose of IFN-α2b rendered the addition of DTIC irrelevant. Furthermore, the analysis of the vaccine-specific immune response showed no differences between the two treatment arms, indicating that the immune adjuvanticity of 3 MU IFN-α2b + DTIC is similar to that of 6 MU IFN-α2b alone.

Because no significant differences were observed between arm 1 and arm 2 patients either in terms of clinical outcome or of Melan-A–specific immune responses, the immunological analyses were conducted cumulatively in arm 1 and arm 2 patients. Overall, our combination strategies enhanced the vaccine-specific CD8^+^ T cell frequencies in ~69% of patients, but this rise did not correlate with the patient clinical outcome [similar to results obtained by Butterfield et al. (44)]. On the contrary, the investigation of the quality of the immune response, carried out by means of a functional multiparameter assay (34), showed that the polyfunctionality of Melan-A–specific T cells is associated with disease control. Indeed, an increase of Melan-A–specific T cells producing simultaneously two and three functions at T92 (i.e., after nine vaccination doses) was observed only in patients who did not relapse thereafter. Similarly, the production of IL-2 by activated Melan-A–specific T cells 4 months after treatment onset positively correlated with the patient clinical outcome, suggesting that these cells are skewed toward a central memory phenotype, which is characterized by high levels of IL-2 production and proliferative ability.

Taking into account the pleiotropic effect of IFN-I (21), in the present study we analyzed not only the vaccine-specific immune responses, like in most immunotherapy clinical trials, but also the modulation of innate immunity. In particular, we focused on NK cells, because studies in IFN-α receptor (IFNAR)–deficient mice demonstrated that IFN-I plays an important role for NK maturation and cytotoxic activity and NK-mediated antitumor effect (22).

Natural killer cells can be distinguished in different subsets based on surface density of CD56 and CD16 (FCγ receptor III) (49). CD56^bright^ NK cells, characterized by high proliferative potential and low cytotoxic ability, are immediate precursors of less proliferating CD56^dim^ NK cells, in which the expression of CD16 increases along with cytotoxicity (49). In our study, an expansion of CD56^dim^CD16^neg^ NK-producing CD107 was observed in nonrelapsing patients following treatment. Of note, these cells exhibit an intermediate maturation level (in terms of proliferating and cytotoxic activity) and are believed to be responsible for natural cytotoxicity against tumor targets.

Overall, findings from this study suggest that the combination of peptide-based vaccination and IFN-α2b can represent a valuable, nontoxic, and nonexpensive strategy to prevent relapse in stage III/IV resected melanoma patients, which may deserve further controlled clinical studies. Mechanistic studies suggest that this novel therapeutic strategy acts through the induction of both adaptive and innate immunity, that is, of polyfunctional Melan-A–specific CD8^+^ T cells and NK cells.

Interferons are currently considered by the majority of clinicians as “old drugs” replaced by the new emerging therapies. However, some novel and promising therapeutic opportunities from new insights stemming from the most recent progress on IFN and cancer research have recently been underlined (8, 21, 50). Of note, the use of old drugs for either new therapeutic uses or with qualitatively new modalities and rationales may exhibit advantages in terms of costs and impact on public health systems (51, 52), as the expenses and time needed for their full clinical development are much lower with respect to those necessary for the all process from drug discovery to the registration of new drugs. We believe that further studies, based on the use of cancer vaccines, together with a local and transient IFN-α treatment and/or IFN-α alone (administered according to our original dosage and schedule), in combination with subsequent CPI administration, can open new perspectives for recurrence prevention in melanoma as well as in other malignant diseases.

## Data Availability Statement

The raw data supporting the conclusions of this article will be made available by the authors, without undue reservation, to any qualified researcher.

## Ethics Statement

The studies involving human participants were reviewed and approved by Regina Elena Cancer Institute ethics committee. The patients/participants provided their written informed consent to participate in this study.

## Author Contributions

FM, VF, PN, IC, FB, and EP designed the study. IC, EP, and FB wrote the clinical protocol. VF, CN, AT, CC, and FC provided and managed patients. BP, PN, IM, FU, and FM developed the methodologies. VF, CN, AT, BP, IM, FU, CB, LC, SB, and NG acquired data. AP and AL prepared drugs and vaccines in the hospital pharmacy. GV performed blood tests. PP, DG, FU, IM, and FM performed statistical analyses and data interpretation. MS, PR, and FU prepared eCRF. FU, VF, IM, PN, BP, FM, FB, and EP wrote or reviewed the manuscript. All authors approved the final version of the manuscript.

### Conflict of Interest

The authors declare that the research was conducted in the absence of any commercial or financial relationships that could be construed as a potential conflict of interest.

## Abbreviations

IFN: Interferon
RFS: relapse-free
OS: overall survival
CPIs: checkpoint inhibitors
IFN-I: type I interferons
TH: T helper
DC: dendritic cell
NK: natural killer
DTIC: Melan-A/MART-1 Melan-A, dacarbazine
ISS: Istituto Superiore di Sanità
IRE: Regina Elena National Cancer Institute
AJCC: American Joint Commission on Cancer
CT: computed tomography
eCRF: electronic case report forms
CTCAE: Common Terminology Criteria for Adverse Events
PE: phycoerythrin
FITC: fluorescein isothiocyanate
SEB: staphylococcal enterotoxin B
IL: interleukin
TNF: tumor necrosis factor
SE: standard error
NED: no evidence of disease
FDA: Food and Drug Administration
EMA: European Medicines Agency
TAAs: tumor-associated antigens.
