# Supplementary material for: Clinical and Immunological Outcomes in High-Risk Resected Melanoma Patients Receiving Peptide-Based Vaccination and Interferon Alpha, With or Without Dacarbazine Preconditioning: A Phase II Study
Source: Front Oncol. 2020 Mar 6;10:202. doi: 10.3389/fonc.2020.00202 (PMC7069350; doi:10.3389/fonc.2020.00202)
Supplement: Supplementary file 1 [file Table_1.DOCX]

**Supplementary Table S1. Single patient characteristics**

| **Patient randomization number** | **Treatment** | **Sex** | **Age (years)** | **Primary tumor site** | **AJCC^d^ stage** | **Breslow index, mm** | **Ulceration** | **RFS^e^ (months)** | **Relapse** | **OS^f^ (months)** | **Death** | **Status at last follow-up** |
| --- | --- | --- | --- | --- | --- | --- | --- | --- | --- | --- | --- | --- |
| 001 | IFN**^a^**-Vaccine | M | 46 | trunk | IIIc | 2,5 | NA | 90 | No | 90 | No | NED**^g^** |
| 002 | DTIC**^b^**-IFN-Vaccine | F | 42 | NA**^c^** | IIIc | NA | NA | 100 | No | 100 | No | NED |
| 003 | DTIC-IFN-Vaccine | M | 52 | temporal region | IIIc | 6,5 | Yes | 11 | Yes | 28 | Yes | Dead |
| 004 | IFN-Vaccine | M | 44 | trunk | IV M1a | 1 | Yes | 102 | No | 102 | No | NED |
| 005 | IFN-Vaccine | M | 59 | NA | IV M1a | NA | NA | 50 | No | 50 | No | NED |
| 006 | DTIC-IFN-Vaccine | M | 23 | calf | IIIc | 3 | Yes | 8 | Yes | 23 | Yes | Dead |
| 007 | IFN-Vaccine | F | 52 | shoulder | IIIc | 2,2 | Yes | 91 | No | 91 | No | NED |
| 008 | DTIC-IFN-Vaccine | F | 34 | NA | IV M1a | NA | NA | 94 | No | 94 | No | NED |
| 009 | DTIC-IFN-Vaccine | M | 66 | trunk | IIIc | NA | No | 81 | No | 81 | No | NED |
| 010 | IFN-Vaccine | M | 68 | scalp | IV M1a | NA | NA | 92 | No | 92 | No | NED |
| 011 | DTIC-IFN-Vaccine | F | 44 | calcaneus | IIIc | 5,5 | No | 13 | Yes | 92 | No | NED |
| 012 | IFN-Vaccine | M | 80 | thigh | IIIc | 2,2 | NA | 4 | Yes | 14 | No | AWD**^h^** |
| 013 | DTIC-IFN-Vaccine | M | 43 | knee | IIIc | 2,8 | NA | 4 | Yes | 7 | Yes | Dead |
| 014 | IFN-Vaccine | F | 40 | auricle | IIIb | NA | NA | 7 | Yes | 32 | No | AWD |
| 015 | IFN-Vaccine | M | 62 | hand finger | IIIc | 4 | Yes | 9 | Yes | 58 | No | AWD |
| 016 | DTIC-IFN-Vaccine | F | 28 | hilum node | IV M1a | NA | NA | 9 | Yes | 79 | No | AWD |
| 017 | DTIC-IFN-Vaccine | M | 51 | eye | IV M1b | NA | NA | 81 | No | 81 | No | NED |
| 018 | IFN-Vaccine | M | 55 | trunk | IIIc | 4,12 | Yes | 1 | Yes | 9 | Yes | Dead |
| 019 | DTIC-IFN-Vaccine | M | 71 | thorax | IIIc | 7,5 | Yes | 3 | Yes | 4 | Yes | Dead |
| 020 | IFN-Vaccine | F | 53 | arm | IV M1a | 1,2 | Yes | 75 | No | 75 | No | NED |
| 021 | DTIC-IFN-Vaccine | M | 70 | trunk | IV M1b | 9,5 | NA | 4 | Yes | 4 | No | AWD |
| 022 | IFN-Vaccine | M | 57 | lumbar region | IIIc | 51 | No | 18 | Yes | 40 | Yes | Dead |
| 023 | IFN-Vaccine | F | 56 | trunk | IIIb | 11 | Yes | 3 | Yes | 13 | Yes | Dead |
| 024 | DTIC-IFN-Vaccine | M | 46 | scapula | IV M1a | 1,75 | Yes | 4 | Yes | 74 | No | AWD |
| 025 | DTIC-IFN-Vaccine | F | 52 | armpit | IV M1a | NA | NA | 4 | Yes | 16 | Yes | Dead |
| 026 | IFN-Vaccine | F | 46 | arm | IV M1b | 1,2 | NS | 70 | No | 70 | No | NED |
| 027 | DTIC-IFN-Vaccine | M | 44 | submammary region | IIIb | 3 | Yes | 52 | No | 52 | No | NED |
| 028 | IFN-Vaccine | F | 46 | lumbar region | IIIc | 1,9 | Yes | 78 | No | 78 | No | NED |
| 029 | DTIC-IFN-Vaccine | F | 29 | scapula | IIIb | 1,4 | NA | 58 | No | 58 | No | NED |
| 030 | IFN-Vaccine | M | 66 | subclavicle region | IIIa | NA | NA | 77 | No | 77 | No | NED |
| 031 | IFN-Vaccine | M | 57 | thorax | IV M1a | NA | NA | 4 | Yes | 15 | Yes | Dead |
| 032 | DTIC-IFN-Vaccine | F | 73 | leg | IIIc | 5 | NA | 4 | Yes | 37 | No | AWD |
| 033 | IFN-Vaccine | M | 52 | abdomen | IIIc | NA | NA | 10 | Yes | 31 | Yes | Dead |
| 034 | DTIC-IFN-Vaccine | M | 67 | armpit node | IIIc | NA | NA | 1 | Yes | 9 | Yes | Dead |

^a^IFN, Interferon-α; ^a^DTIC, Dacarbazine; ^c^NA, not avaliable; ^d^AJCC, American Joint Committee on cancer; ^e^RFS, relapse-free survival; ^f^OS, overall survival; ^g^NED, alive and no evidence of disease; ^h^AWD, alive with disease.
