# Supplementary material for: Clinical and Immunological Outcomes in High-Risk Resected Melanoma Patients Receiving Peptide-Based Vaccination and Interferon Alpha, With or Without Dacarbazine Preconditioning: A Phase II Study
Source: Front Oncol. 2020 Mar 6;10:202. doi: 10.3389/fonc.2020.00202 (PMC7069350; doi:10.3389/fonc.2020.00202)
Supplement: Supplementary file 2 [file Table_2.DOCX]

**Supplementary Table S2. Flow-cytometry reagents details**

| **Staining panel** | **Antigen/Ligand** | **Clone** | **Fluorochrome** | **Species** | **Manufacturer** | **Nationality** |
| --- | --- | --- | --- | --- | --- | --- |
| FMT - total CD8^+^ cells and MART-1 tetramer^+^ CD8^+^ cells functionality | CD107a | H4A3 | FITC | mouse | BD Pharmingen | California |
|  | MART-1 Tetramer | NA | PE | NA | Beckman Coulter | California |
|  | TNF-α | Mab11 | PerCP Cy5.5 | mouse | E-Bioscience | Massachussetts |
|  | IFN-γ | B27 | PE Cy7 | mouse | BD Pharmingen | California |
|  | IL-2 | MQ1-17H12 | APC | rat | BD Pharmingen | California |
|  | CD8 | SK1 | APC H7 | mouse | BD Pharmingen | California |
| NK subpopulations and NKT cells phenotype and functionality | CD107a | H4A3 | FITC | mouse | BD Pharmingen | California |
|  | CD56 | B159 | PE | mouse | BD Pharmingen | California |
|  | CD3 | SP34-2 | PerCP Cy5.5 | mouse | BD Pharmingen | California |
|  | IFN-γ | B27 | PE Cy7 | mouse | BD Pharmingen | California |
|  | CD16 | LNK16 | Alexa Fluor 647 | mouse | Serotec | California |
|  | Dead cells | NA | nIR | NA | Molecular Probes | Oregon |
