# Supplementary material for: Clinical and Immunological Outcomes in High-Risk Resected Melanoma Patients Receiving Peptide-Based Vaccination and Interferon Alpha, With or Without Dacarbazine Preconditioning: A Phase II Study
Source: Front Oncol. 2020 Mar 6;10:202. doi: 10.3389/fonc.2020.00202 (PMC7069350; doi:10.3389/fonc.2020.00202)
Supplement: Supplementary file 3 [file Presentation_1.PPTX]

## Slide 1
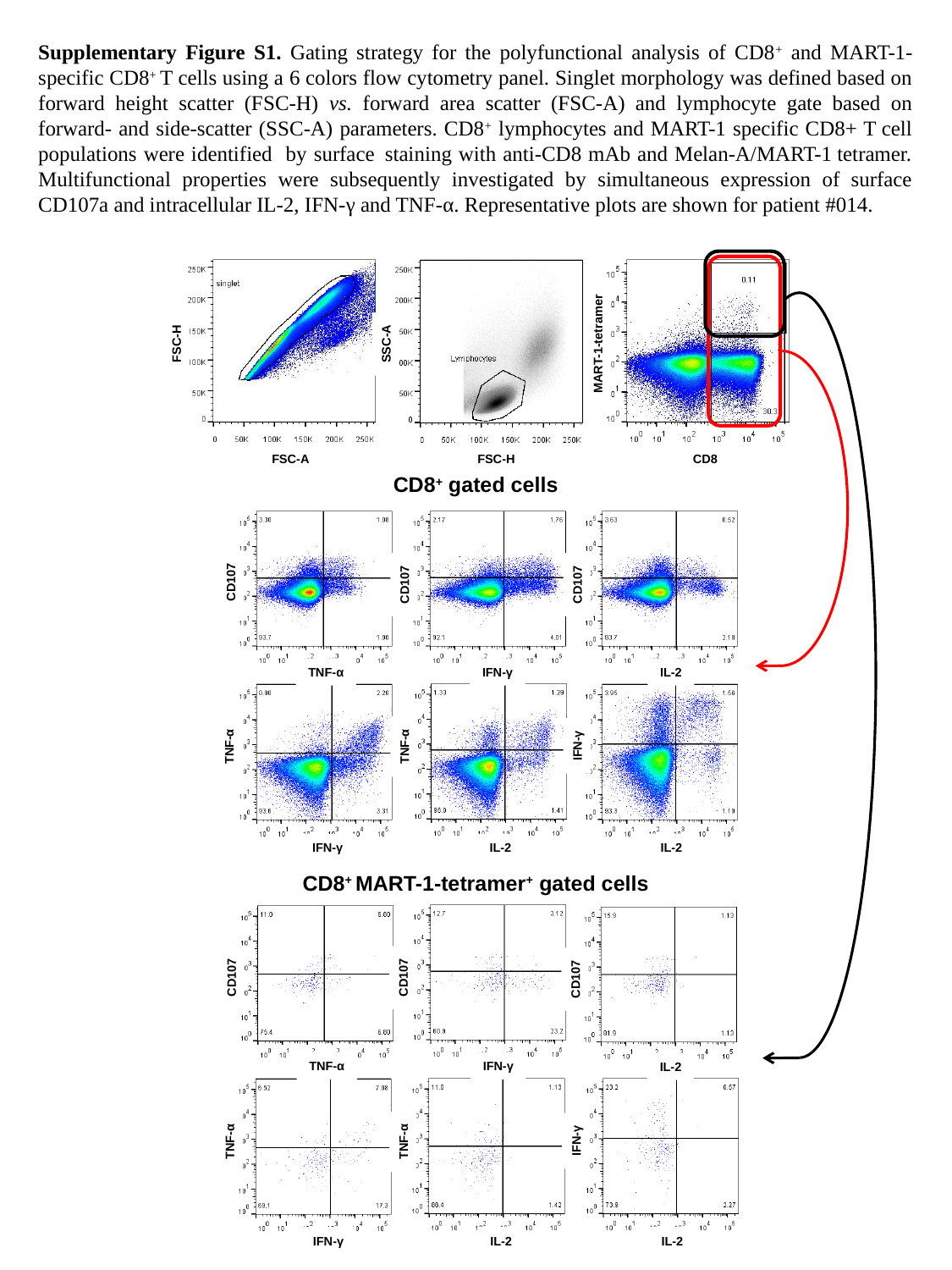

Supplementary Figure S1. Gating strategy for the polyfunctional analysis of CD8+ and MART-1-specific CD8+ T cells using a 6 colors flow cytometry panel. Singlet morphology was defined based on forward height scatter (FSC-H) vs. forward area scatter (FSC-A) and lymphocyte gate based on forward- and side-scatter (SSC-A) parameters. CD8+ lymphocytes and MART-1 specific CD8+ T cell populations were identified by surface  staining with anti-CD8 mAb and Melan-A/MART-1 tetramer. Multifunctional properties were subsequently investigated by simultaneous expression of surface CD107a and intracellular IL-2, IFN-γ and TNF-α. Representative plots are shown for patient #014.
FSC-H
SSC-A
MART-1-tetramer
FSC-A
FSC-H
CD8
CD8+ gated cells
CD107
CD107
CD107
TNF-α
IFN-γ
IL-2
IFN-γ
TNF-α
TNF-α
IFN-γ
IL-2
IL-2
CD8+ MART-1-tetramer+ gated cells
CD107
CD107
CD107
TNF-α
IFN-γ
IL-2
IFN-γ
TNF-α
TNF-α
IFN-γ
IL-2
IL-2
