# Supplementary material for: Clinical and Immunological Outcomes in High-Risk Resected Melanoma Patients Receiving Peptide-Based Vaccination and Interferon Alpha, With or Without Dacarbazine Preconditioning: A Phase II Study
Source: Front Oncol. 2020 Mar 6;10:202. doi: 10.3389/fonc.2020.00202 (PMC7069350; doi:10.3389/fonc.2020.00202)
Supplement: Supplementary file 4 [file Presentation_2.PPTX]

## Slide 1
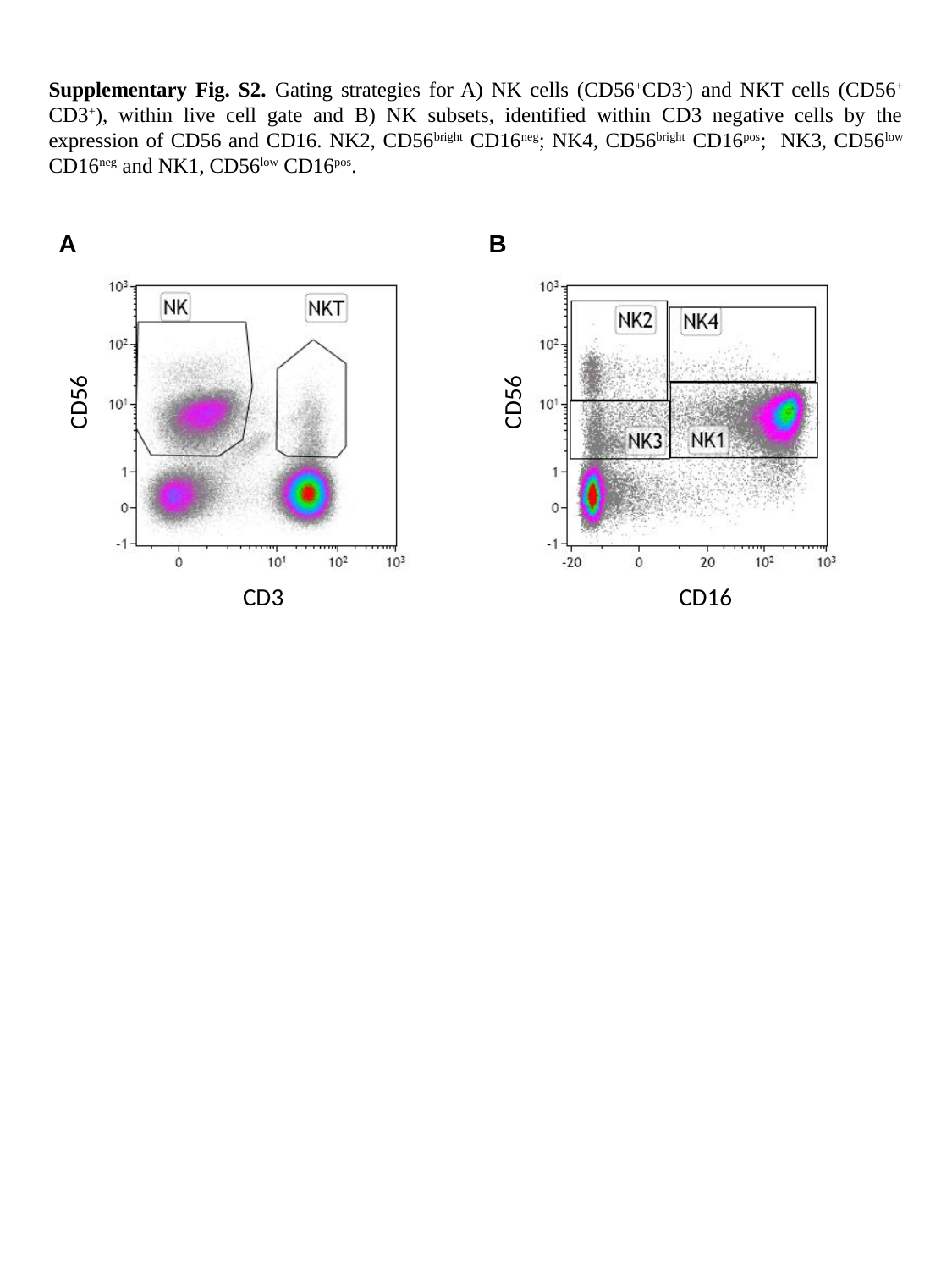

Supplementary Fig. S2. Gating strategies for A) NK cells (CD56+CD3-) and NKT cells (CD56+ CD3+), within live cell gate and B) NK subsets, identified within CD3 negative cells by the expression of CD56 and CD16. NK2, CD56bright CD16neg; NK4, CD56bright CD16pos; NK3, CD56low CD16neg and NK1, CD56low CD16pos.
A
B
CD56
CD56
CD3
CD16
