# Supplementary material for: Clinical and Immunological Outcomes in High-Risk Resected Melanoma Patients Receiving Peptide-Based Vaccination and Interferon Alpha, With or Without Dacarbazine Preconditioning: A Phase II Study
Source: Front Oncol. 2020 Mar 6;10:202. doi: 10.3389/fonc.2020.00202 (PMC7069350; doi:10.3389/fonc.2020.00202)
Supplement: Supplementary file 5 [file Presentation_3.PPTX]

## Slide 1
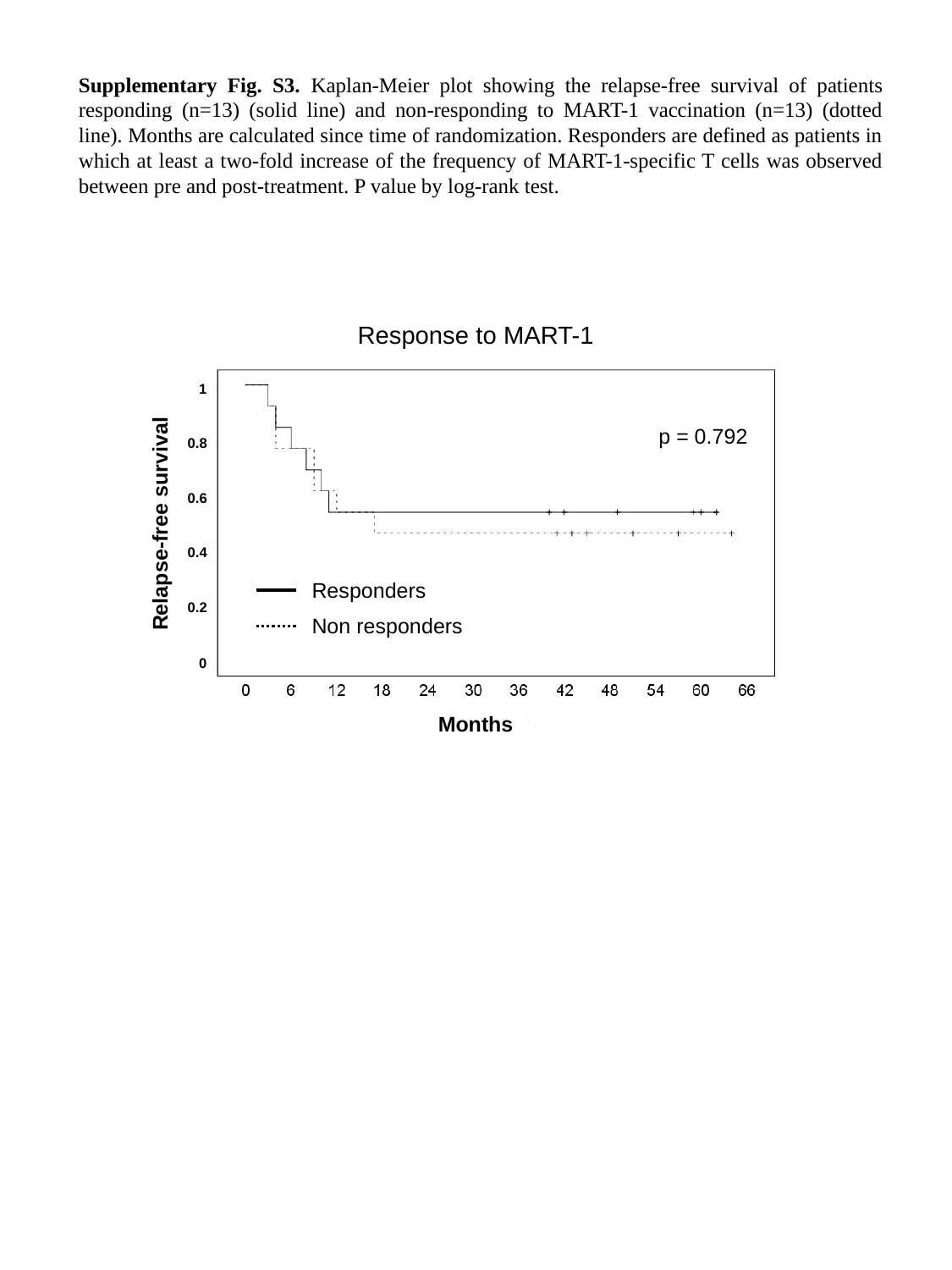

Supplementary Fig. S3. Kaplan-Meier plot showing the relapse-free survival of patients responding (n=13) (solid line) and non-responding to MART-1 vaccination (n=13) (dotted line). Months are calculated since time of randomization. Responders are defined as patients in which at least a two-fold increase of the frequency of MART-1-specific T cells was observed between pre and post-treatment. P value by log-rank test.
Response to MART-1
1
p = 0.792
0.8
0.6
Relapse-free survival
0.4
Responders
0.2
Non responders
0
Months
